# Promoting prudent use of antibiotics: the experience from a multifaceted regional campaign in Greece

**DOI:** 10.1186/1471-2458-14-866

**Published:** 2014-08-22

**Authors:** Diamantis Plachouras, Anastasia Antoniadou, Efthymia Giannitsioti, Lambrini Galani, Ioannis Katsarolis, Dimitra Kavatha, George Koukos, Periklis Panagopoulos, Antonios Papadopoulos, Garyphalia Poulakou, Vissaria Sakka, Maria Souli, Styliani Sybardi, Sotirios Tsiodras, Kyriaki Kanellakopoulou, Helen Giamarellou

**Affiliations:** 4th Department of Internal Medicine, University Hospital « Attikon », 1 Rimini Avenue, 12462 Haidari Athens, Greece; Department of Internal Medicine, Thriassio General Hospital, Elefsina, Greece; 6th Department of Internal Medicine, Hygeia General Hospital, Marousi Athens, Greece

**Keywords:** Antimicrobial consumption, Academic detailing, Public education

## Abstract

**Background:**

Antibiotic resistance, a major public health problem, has been linked to antibiotic consumption. In Greece both consumption and resistance rates are among the highest in Europe. A multifaceted campaign targeting both physicians and parents of school children was implemented for the first time in order to educate the public and update doctors, aiming to promote judicious use of antibiotics and hopefully decrease its consumption.

**Methods:**

The programme consisted of a public education campaign and academic detailing of primary care physicians in the district of Corinth in Peloponnese. The experience and perceptions of parents were recorded in the meetings in the form of course evaluation and assessment, anonymous questionnaires. The use of Rapid Antigen Detection Test (RADT) for streptococcal pharyngitis by primary care physicians was also assessed by use of anonymous questionnaires. Antibiotic consumption was compared before and after the programme between the district of Corinth and the other districts of Peloponnese, as well as at a national level.

**Results:**

Antibiotic consumption remained unaltered at 26 Defined daily doses per 1000 Inhabitants per Day (DID) in accordance with the trend in other regions and at a national level. However, the utilization of Amoxycillin and Penicillin was increased by 34.3%, while the use of other antimicrobial classes including macrolides, cephalosporins and fluoroquinolones decreased by 6.4-21.9%. The use of RADT did not lead to a significantly decreased antimicrobial consumption.

**Conclusions:**

A multifaceted educational programme targeting both the general public and primary care physicians was associated with rationalization in the choice of antimicrobial. A reduction in the total antimicrobial consumption was not achieved.

**Electronic supplementary material:**

The online version of this article (doi:10.1186/1471-2458-14-866) contains supplementary material, which is available to authorized users.

## Background

Antibiotic resistance is an increasing problem of public health. There is an association between antibiotic resistance and antibiotic consumption [1]. In Europe there are significant differences between northern and southern countries in antibiotic consumption with parallel differences in resistance. Greece is among the countries with the highest consumption and resistance rates among European Countries [2, 3].

Antibiotic consumption is highest in the community compared to hospitals and is especially associated with upper respiratory tract infections during the winter months [4]. The great majority of these prescriptions are not necessary, due to the viral cause in the majority of these infections. Therefore antibiotic overuse in the community can be targeted without any health hazards.

A number of interventions has been implemented with variable success [5]. These interventions have taken various forms and are targeted either to the public, including education and media campaigns, or to physicians in the form of academic detailing among others alone or in combination. The outcome of these interventions has been difficult to assess due to the multiple confounding factors that affect antibiotic use, including seasonal and annual variability in the incidence of infections. However, a majority of the studies indicated a reduction in the antibiotic prescription rates following the intervention [3].

Based on the above data a multifaceted regional campaign was performed targeting both the public and primary care physicians in the region of Corinth in Greece in 2009–10.

## Methods

### Study design

The educational material and the programme were organized by the Medical School of the University of Athens in cooperation with the Prefecture of Corinth and the Medical Association of Corinth in Peloponnese. It consisted of a public education campaign and academic detailing of the primary care physicians in the district of Corinth (Jan-Feb 2009) and an assessment of the use of Rapid Antigen Detection Test (RADT) for *Streptococcus pyogenes* group A by the primary care providers (Feb-Apr 2010).

### Public education

Seventeen two-hourly educational meetings were organized in all major municipalities of the district with parents of children in nursing care and primary school. The title of the meetings was “What Do Greek Parents Need to Know on the Proper Use of Antibiotics”. Before the meeting parents were asked to fill an anonymous questionnaire for their experience and perceptions on the use of antibiotics in common clinical scenarios (Additional file 1). In every meeting there was a short introductory lecture by a physician specialized in infectious diseases. Following this, parents were given an educational pamphlet on the use of antibiotics for common infections in the community, published by the Institute of Pharmaceutical Research and Technology (IFET) and available for download at http://www.ifet.gr/antibiotics_site/FrameSet6.htm. An open discussion in the form of question-and-answer followed between parents and experts. The only mass media intervention in the present campaign was a press-conference organized by the prefecture for the local media at the start of the intervention. Filling the questionnaire by parents was on a voluntary basis and anonymous, in the scope of providing a background for the discussion to follow and the issues raised by the parents.

### Academic detailing

This was carried out in the form of meetings with the physicians who provide primary care for patients with respiratory tract infections including general practitioners, pediatricians, otorhinolaryngologists and chest physicians. The meetings included a lecture entitled “Bugs and antibiotics: the role of the Greek physician in the fight against microbial resistance” followed by four short interactive sessions based on the management of specific cases. Such cases included patients with acute pharyngitis, otitis media and urinary tract infections. The meetings were organized under the auspices of the local Medical Association. The use of bedside RADT for *Streptococcus pyogenes* was discussed with the participating physicians. Participating physicians also received a booklet on the management of community acquired infections edited by the National Organization for Medicines (EOF) and another with the guidelines of the Greek Center for Disease Control and Prevention (KEELPNO) on the treatment of infections (also available to download at http://www.keelpno.gr, http://www.eof.gr and http://www.ifet.gr).

In addition, five meetings were arranged with primary care physicians at the five public primary health care centers. These meetings were based on the same programme with the first meeting. Another meeting was arranged with district dentists under the auspices of the local Dental Association. This meeting was targeted on the use of antibiotics by dentists including endocarditis prophylaxis.

In January 2010 primary care physicians in the region including both pediatricians and general practitioners were invited via the local medical association to share their experience with use of RADT test and the management of patients with pharyngo-tonsillitis. Physicians volunteering to participate would fill-in an anonymous form with details on Centor criteria [6], the use of RADT, the results and the antimicrobials prescribed, if any (anonymity referred to both doctor and case details). The form had been previously implemented in another study [7]. The end of the recording was in April 2010 paralleling the winter season with the highest rate of upper respiratory infections in Greece.

### Ethics

All questionnaires used in the campaign were anonymous. The questionnaires filled for the RADT use survey were also anonymous and had been subjected to approval by the Ethics Committee of the University General Hospital ATTIKON [7]. The survey for the use of RADT was performed under the approval of the regional Medical Association of Corinth. The public campaign was approved and took place under the auspices of the Prefecture of Corinth and was designed and executed in compliance with the Helsinki Declaration.

### Data analysis

Antibiotic consumption data was kindly provided by IMS, Greece for the district of Corinth, the other districts of Peloponnese and on a national level. Only antibiotics prescribed for respiratory tract infections were included in the study. These were phenoxymethylpenicillin, amoxicillin and the combination of amoxicillin and clavulanate, 1st and 2nd generation cephalosporins, macrolides and the fluoroquinolones levofloxacin and moxifloxacin. Defined daily doses (DDD) per 1000 inhabitants per day (DID) were calculated based on the ATC/DDD index of the WHO collaborating Centre for Drug Statistics Methodology. The rest of districts in Peloponnese and the national rate served as controls and data were compared before and after the campaign between January 2009 and February 2009 with Fisher’s exact test. In the RADT-related questionnaire, the prescription rate of antibiotics was compared with Fisher’s exact test between those reporting using the RADT vs. those that did not.

## Results

In total 772 parents participated in the meetings (approximately 7% of the estimated number in total, if a 1:1 child: parent ratio is considered - a total of approx. 11000 children attended primary school and kindergartens in the prefecture of Corinth in 2009). In the academic detailing 111 out of 486 physicians in the prefecture (approximately 20%) and 30 out of 151 dentists (approximately 23%) were present (data derived from http://www.statistics.gr for the year 2009).

### Parent questionnaires

In total 293 parent/care giver responses were collected (38% of attendees). Demographic data and responses are described in Table 1.

**Table 1:** **Responses of parents/care givers to questionnaire**

	No (%)
Female	228 (77.8)
**Education**	
Higher	136 (46.4)
Higher secondary	79 (27)
Secondary	42 (14.3)
Primary	15 (5.1)
Children <6 years old in household	109 (37.2)
Might use antibiotics without physician consultation	26 (8.9)
Stocked antibiotics at home	94 (32.1)
**Reason for antibiotic prescription**	
Purulent throat	62 (1.2)
Ear pain	34 (11.6)
Combination of above	43 (14.7)
Fever	50 (17)
Discoloured sputum	17 (5.8)
**In case the physician did not prescribe an antibiotic**	
Accept the advice	261(89.1)
Consult another physician	15 (5.1)
Buy antibiotic without prescription	7 (2.4)
Antibiotic use in previous six months	68 (23.2)
Antibiotic use of children in previous six months	87 (29.7)
Occasionally buying antibiotics from pharmacy without prescription	34 (11.6)
Experienced adverse events by antibiotics*	96 (32.8)

*Adverse events reported: 31 (35.6%) rash, 26 (29.9%) allergic reaction, 16 (18.4%) diarrhea and 14 (16%) combination of these.

### Antibiotic consumption

**Figure 1:**
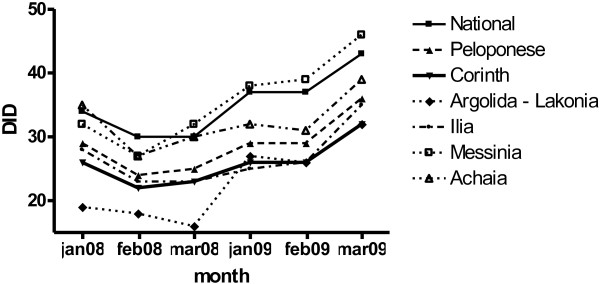
**Trend of antibiotic utilization during the winter months of 2008 and 2009 on a national and regional level in the districts of Peloponnese.**

**Figure 2:**
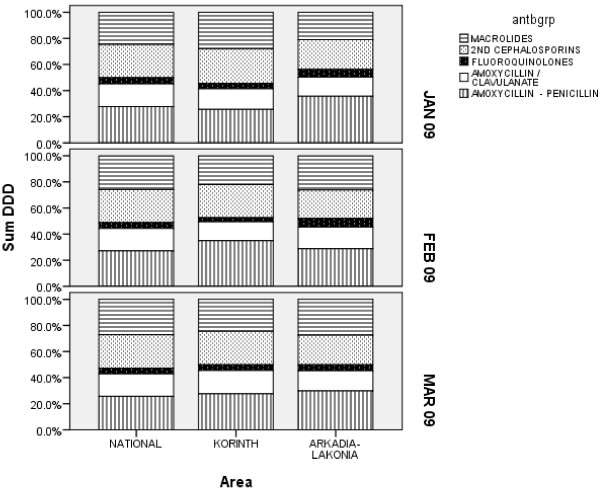
**Relative utilization of various antibiotic groups before and after the intervention in the district of Corinth compared to the National rate and the neighbouring districts of Lakonia and Arkadia.**

Antibiotic utilization in the region of Corinth was unchanged in January and February 2009 at 26 DID and increased to 32 DID in March 2009. This trend reflected the overall trend at a national level as well as at a regional level in the neighboring districts where no intervention was implemented (Figure 1). However, when the utilization of antibiotic groups was compared it appeared that in the district of Corinth there was a 34,3% increase in the use of Amoxycillin and Penicillin, while the utilization of macrolides, 2nd generation cephalosporins, fluoroquinolones and amoxicillin clavulanate decreased 21.9%, 6.4%, 21.9% and 9.4% respectively (*p* = 0.02) (Figure 2 and Table 2). On the other hand, no significant change in the antibiotic distribution was observed in the same time period at the national utilization rates. In addition, in the neighboring districts of Arkadia and Lakonia the use of macrolides, fluoroquinolones and amoxicillin/clavulanate increased by 22.7%, 12.6% and 10.3% respectively and the use of amoxicillin/penicillin and of 2nd generation cephalosporins decreased by 20.9% and 7.4% respectively (Figure 2). In March 2009, however, antibiotic use in the district of Corinth reverted to previous levels and antibiotic group percentages approached again the national rates.

**Table 2:** **Utilization of various antibiotic groups (DDD) before and after the intervention in the district of Corinth compared to the neighbouring districts of Lakonia and Arkadia**

	Corinth		Lakonia–Arkadia	
	Jan 09	Feb 09	Jan 09	Feb 09
**Macrolides**	56753	44332	33297	40865
**2nd gen Cephalosporins**	53963	50464	36296	33601
**Fluoroquinolones**	8765	6449	9213	10374
**Amoxycillin/clavulanate**	32031	29013	23375	25790
**Amoxycillin/penicillin**	52343	70313	56801	44905

### RADT use

Thirteen physicians provided questionnaires for the use of RADT study, with a total number of 270 patient cases. Eleven physicians - 8 pediatricians, 2 otorhinolaryngologists and 1 general practitioner- reported consistent use of RADT group. They contributed 216 (80%) cases questionnaires. Two physicians – one general practitioner and one pediatrician - reported an empirical approach without RADT treatment group and contributed 54 (20%) cases questionnaires. In the RADT group 177 (85.1%) patients and in the empirical treatment group 34 (64.2%) patients were children (p < 0.001). The utilization of antibiotics in the two groups is presented in Table 3. Reasons for administration of antibiotics despite a negative RADT are described in Table 4. Administration of antibiotics according to Centor scale in both groups is depicted in Table 5. Throat swab culture was performed in only 16 (5.9%) cases, among which 4 were positive.

**Table 3:** **Antibiotic prescription in the groups of management based on RADT and empirical treatment**

	Empirical treatment	Treatment based on RADT	***p***value
Antibiotic prescribed – No (%)	21 (38.9)	97 (44.9)	*0.44*
Positive RADT – No (%)		63 (29.2)	
Antibiotic prescribed with negative RADT		34 (22.4)	
**Antibiotic prescribed**			
Penicillin	0 (0)	11 (11.5)	*0.0001*
Amoxycillin	2 (9.5)	31 (32.3)	
Amoxicillin/clavulanate	3 (14.3)	10 (10.4)	
Macrolide	12 (57.1)	34 (35.4)	
Cephalosporin	4 (19)	10 (10.4)

**Table 4:** **Stated reasons for prescription of antibiotics in patients with negative RADT**

Reason	No (%)
Clinical picture consistent with streptococcal infection*	23 (67)
Otitis	3 (9)
Positive culture	2 (6)
Bronchitis	2 (6)
Technical problem with RADT	1 (3)
Culture result pending	1 (3)
Patient already self-medicated	1 (3)
Leukocytosis	1 (3)

*Only in 14 of these patients the Centor score was >1. In four patients there was a rash reminiscent of scarlet fever.

**Table 5:** **Number (%) of patients prescribed an antibiotic according to Centor score in the empirical and RADT-based groups**

	Centor score				
	0	1	2	3	4
Empirical	1 (12.5)	4 (30.8)	5 (25.0)	7 (77.8)	4 (100)
RADT-based	4 (18.2)	15 (25.9)	29 (46.8)	34 (65.4)	14 (66.7)

## Discussion

Antibiotic resistance is an emerging public health issue of major concern worldwide which has been associated with antibiotic consumption [1, 8]. The majority of antibiotics are prescribed in outpatients for upper respiratory tract infections, which are usually of viral origin [4, 9]. Therefore, a great proportion of prescribed antibiotics is unnecessary and provide only selection pressure for the emergence of resistant bacteria. Antibiotic consumption is a complex issue influenced by multiple factors, including attitudes, knowledge and behavior of prescribers and patients, as well as the pharmaceutical industry. Interventions targeting a single aspect affecting antibiotic use have in the majority of cases failed to achieve significant changes in antibiotic consumption. Multifaceted interventions are usually more successful but also more costly and organizationally challenging [10].

The present campaign was designed to address antibiotic consumption in upper respiratory tract infections in a primary care setting during the winter months of 2009 and 2010, when the majority of unnecessary antibiotics are usually prescribed. It included initiatives targeting the public and specifically parents of young children, as well as academic detailing of health care providers in the primary care sector. Academic detailing (or “educational outreach”) is effective at inducing change in prescribing behavior in various settings [11].

Antibiotic utilization behavior in the target population was assessed by the use of a questionnaire addressing the main public behaviors associated with antibiotic overuse. In accordance with previous studies in both in Greece [12] and in other countries [13, 14] the majority of antibiotics are prescribed for upper respiratory tract infections while a significant minority of the public use antibiotics without prescription and may stock antibiotics at home. These data confirm that there is a significant gap between academic knowledge and everyday prescription practice and patient behavior regarding antibiotics. It must be stressed that antibiotics are prescription-only drugs in Greece. However this is not strictly enforced, potentially leading to over the counter antimicrobial sales by pharmacies.

According to the results of the study no major change in the total number of antibiotics prescribed was detected. However the observed data indicate that in the targeted district antibiotic utilization was more rational, as reflected by significantly increased use of amoxicillin or penicillin according to the national guidelines and a decrease in the use more broad-spectrum antimicrobials like macrolides, 2nd generation cephalosporins, fluoroquinolones and amoxicillin/clavulanate. However, after the end of the campaign, antibiotic use tended to revert to previous levels, indicating the need for continuous educational initiatives (or at least of longer duration or repetitive).

One of the limitations of the analysis of the data is the before-after design with comparisons on the months before and after the intervention. Such analyses do not account for seasonal or other temporal confounding factors. A time-series analysis might be more informative. However, data were available only for limited time points, preventing time-series analysis. A further limitation that may have affected the effectiveness of the intervention is the rate of participation of both healthcare professionals and parents. A bias towards increased participation of physicians and parents already familiar with appropriate antibiotic use cannot be excluded either.

It has been shown in various settings that the use of RADT for the detection of streptococcal antigens on pharyngeal swabs improves the diagnostic accuracy of streptococcal pharyngitis compared to the clinical criteria alone, and is comparable to and more practical than the use of pharyngeal swab culture [7, 15, 16]. In addition access to RADT to pediatricians has been associated with a 50% decrease in the use of antibiotics compared to management based on clinical criteria, from 72 to 33%. RADT has been recommended by the guidelines for the management of pharyngitis by the Infectious Disease Society of America [17]. In the present study, physicians providing primary care in both children and adults in everyday clinical practice for the winter season of 2010 had also participated in the educational campaign in the previous year. No difference in the use of antibiotics was observed between those with and those without use of RADT. A variety of reasons may account for this difference from previous studies. It appears that physicians in the present study not using RADT prescribed less antibiotics (38.9%) compared to similar groups in previous studies, indicating perhaps better adherence to clinical indications. The effect of the previous educational campaign cannot be adequately assessed. Another interesting finding is that the most usually cited reason for prescribing antibiotics despite a negative RADT was a clinical picture “consistent with” streptococcal pharyngitis. Culture of a pharyngeal swab was performed in a minority of patients reflecting probably the impracticality of this procedure, because of the time required for the result and the necessity for a second appointment. Pharyngeal swab cultures which are recommended by the IDSA guidelines for certain groups were available in most other studies and may have contributed to the observed higher reduction in the use of antibiotics.

Even though no reduction was observed in the antibiotic prescription rate, there were significant differences in the type of antibiotics prescribed in the two groups. In the RADT group penicillin and amoxicillin, which are the recommended antibiotics according to the guidelines, were prescribed more frequently compared to all other categories. This was accompanied by a significant decrease in the use of both macrolides and cephalosporins, indicating that the use of RADT encourages treatment with more narrow-spectrum antibiotics. The latter finding is especially important in Greece where consumption of macrolides is especially high with accompanying high rates of resistance.

## Conclusions

In conclusion, a multifaceted campaign was performed for the first time in Greece, addressing the multiple factors that influence the utilization of antibiotics (providers and public). In general, it revealed the gap that needs to be filled in the proper education of the public in terms of antibiotic use and it provided a starting point for improvement in certain aspects of providers’ practices in terms of antibiotic prescribing. Similar campaigns implemented for longer period of time and at a larger scale may promote the prudent use of antibiotics, with a final aim to reduce antimicrobial resistance in the community.

## Electronic supplementary material

Additional file 1: **Questionnaire for the evaluation of the parents’ experience, knowledge and behavior concerning antibiotic use.** (DOCX 18 KB)
