# Supplementary material for: Promoting prudent use of antibiotics: the experience from a multifaceted regional campaign in Greece
Source: BMC Public Health. 2014 Aug 22;14:866. doi: 10.1186/1471-2458-14-866 (PMC4148920; doi:10.1186/1471-2458-14-866)
Supplement: Supplementary file 1 — Additional file 1: Questionnaire for the evaluation of the parents’ experience, knowledge and behavior concerning antibiotic use. (DOCX 18 KB) [file 12889_2013_6978_MOESM1_ESM.docx]

**Figure 1.** Questionnaire for the evaluation of the parents' experience, knowledge and behavior concerning antibiotic use

**Age...........**

**Place of residence................................................Education level..............**

**Children in the family < 12 ys of age (circle as appropriate)**

1 2 3 4 5 other ...............................

**Is there a child less than 1 year of age living with you**? yes no

**Is there a child less than 3 years of age living with you?** yes no

**Are there elderly people (>65 ys) living with your family?** yes no

**Do you consult with a doctor before getting an antibiotic?**

always sometimes no I do not wish to answer

**Are there antibiotics in your house pharmacy stocked just in case?**

yes no

**In which of the following cases do you believe that the administration of an antibiotic is necessary?**

"fever"

"ear pain"

"cough"

"dripping nose"

"diarrhea"

"pus in the tonsils"

"in order not to get worse when I have caught a cold"

"to get well sooner when I have a cold"

"nasal/sputum discharge of yellow or green color"

**If the doctor does not prescribe antibiotics, what do you do next?**

I consult with a different doctor

I ask a pharmacist

I get antibiotics on my own

I do not get an antibiotic

**Have you had an antibiotic in the last 6 months?** yes no

**If yes, how many times**? 1 2 -3 more than 3
